# Virtual Avatar for Emotion Recognition in Patients with Schizophrenia: A Pilot Study

**DOI:** 10.3389/fnhum.2016.00421

**Published:** 2016-08-26

**Authors:** Samuel Marcos-Pablos, Emilio González-Pablos, Carlos Martín-Lorenzo, Luis A. Flores, Jaime Gómez-García-Bermejo, Eduardo Zalama

**Affiliations:** ^1^Cartif Foundation, Parque Tecnológico de BoecilloValladolid, Spain; ^2^Research Unit, Hermanas Hospitalarias Centro Sociosanitario PalenciaPalencia, Spain; ^3^ITAP-DISA, University of ValladolidValladolid, Spain

**Keywords:** schizophrenia, facial recognition of emotions, realistic virtual avatar

## Abstract

Persons who suffer from schizophrenia have difficulties in recognizing emotions in others’ facial expressions, which affects their capabilities for social interaction and hinders their social integration. Photographic images have traditionally been used to explore emotion recognition impairments in schizophrenia patients, but they lack of the dynamism that is inherent to facial expressiveness. In order to overcome those inconveniences, over the last years different authors have proposed the use of virtual avatars. In this work, we present the results of a pilot study that explored the possibilities of using a realistic-looking avatar for the assessment of emotion recognition deficits in patients who suffer from schizophrenia. In the study, 20 subjects with schizophrenia of long evolution and 20 control subjects were invited to recognize a set of facial expressions of emotions showed by both the said virtual avatar and static images. Our results show that schizophrenic patients exhibit recognition deficits in emotion recognition from facial expressions regardless the type of stimuli (avatar or images), and that those deficits are related with the psychopathology. Finally, some improvements in recognition rates (RRs) for the patient group when using the avatar were observed for sadness or surprise expressions, and they even outperform the control group in the recognition of the happiness expression. This leads to conclude that, apart from the dynamism of the shown expression, the RRs for schizophrenia patients when employing animated avatars may depend on other factors which need to be further explored.

## Introduction

Schizophrenia is a serious mental disorder that affects about 1% of the population. It is the most expensive mental illness in terms of direct health costs, indirect productivity losses, and the impact on the family and society. There is also a notable impairment of the person performance in areas such as employment, family and social integration (APA, 2002). This disorder is characterized by a variety of symptoms: hallucinations, delusions, affective flattening, disorganized behavior, apathy, social withdrawal, cognitive impairment, and others.

Among those symptoms, social cognition is a major construct to investigate in schizophrenia. Social cognition has been defined as the human ability and capacity of perceiving others’ intention and predisposition (Brothers, 1990). For Green et al. (2005), social cognition refers to the mental operations underlying social interactions, which include processes involved in perceiving, interpreting, and generating responses to the intentions, dispositions, and behaviors of others. In the case of patients with schizophrenia, impairments in social cognition are present before the onset of psychosis, suggesting that social cognition may be a trait marker of the illness. Although there is still a lack of complete consensus about the concrete scope and significance of those impairments, some initiatives have arisen which try to study cognitive impairments in schizophrenia that include social domains. Examples of such studies are the Measurement and Treatment Research to Improve Cognition in Schizophrenia (MATRICS; Green et al., 2000, 2008), and the Cognitive Neuroscience for Treatment Research to Improve Cognition in Schizophrenia (CNTRICS; Carter et al., 2009). Following these frameworks, three major areas of social cognition in Schizophrenia can be considered: the theory of mind, attribution style and the facial recognition of emotions (Penn et al., 2008). The present study focuses on the last of those areas.

### Facial Recognition of Emotions

Concerning emotion recognition, subjects with schizophrenia show a deficit in facial emotion discrimination and identification. Also, this deficit has been observed in other diseases such as depression, mania, dementia, brain injury, autism, etc. (Mandal et al., 1998; Penn et al., 2001; Pinkham et al., 2003).

Several works have focused on the ability of schizophrenic patients to recognize face emotions. Regarding the clinical condition of patients with schizophrenia, the deficit in the facial recognition of emotions is a permanent feature of the disease. This deficit is present from the beginning of the illness and tends to stay in more chronic stages (Kee et al., 2003). Patients have more difficulty to recognize negative emotions such as anger or fear, interpreting them wrongly as neutral expressions (Kohler et al., 2003). In relation to the symptoms, it seems that subjects with delusions of persecution and paranoia have better outcomes than subjects with schizophrenia, but without paranoia (Lewis and Garver, 1995). Patients with negative symptoms would have further deterioration in these tests (Green and Nuechterlein, 1999). With respect to gender, women misinterpret neutral faces as sad ones more often than men, and males reported neutral faces as angry ones (Weiss et al., 2007). The ability of recognizing facial emotions seems to be related to other cognitive functions, as well as to various aspects of social functioning and the quality of life (Kee et al., 2003; Kohler et al., 2010). For example, in Silver et al. (2002) some authors relate social cognition impairments to alterations in the memory, abstract thinking, language processing and attention.

Apart from emotional recognition impairment diagnosis, many psychosocial intervention programs in the field of social cognition (including emotion recognition) have been used for years: Social Skills Training (SST; Liberman et al., 1985), Integrated Psychological Therapy (IPT; Roder et al., 1996) and Training in Emotional Intelligence (TEI; Vauth et al., 2001). Also, specific social cognition treatment techniques have also been developed more recently: Social Cognition and Interaction Training (SCIT; Penn et al., 2005), Social Cognition Enhancement Training (SCET; Choi and Kwon, 2006), MetaKognitives Training (MKT; Moritz and Woodward, 2007) and Training of Affect Recognition (TAR; Wölwer et al., 2005). Most of those studies and treatment procedures focus on individual mechanisms and observational perspectives. However, during the last years, researchers have started to study schizophrenic patients during interactive situations (Park et al., 2011; Lee et al., 2013). The main goal is to make use of virtual environments and avatars to provide new objective methods for assessing patients’ interpersonal behavior characteristics in a social setting.

In terms of emotion recognition, one drawback of current diagnosis and treatment procedures resides in the fact that there is a lack of a methodology that allows correlating the existing methods of encoding and parameterizing facial movements with the different cognitive impairments. To date, several methods have been proposed for facial motion parameterization: Facial Action Coding System (FACS; Ekman and Friesen, 1978; Ekman et al., 2002), Maximally Discriminative Facial Movement Coding System (MAX; Izard, 1983), and AFFEX (Tronick et al., 2002). However, most programs for the identification and treatment of affection recognition disorders in patients with schizophrenia have been based on the use of static images of facial emotions. These images are obtained from different existing facial coding systems, the FACS being one of the most accepted ones. The use of this kind of image results in a hardly-parameterizable identification and treatment process. In addition, static images lack the important information that is inherent to face to face social interactions, so the person under study (either suffering from schizophrenia or not) may not correctly differentiate the actual emotion (Lemay et al., 2000; Wehrle et al., 2000; Adams et al., 2003; Klucharev and Sams, 2004; Joyal et al., 2014). Therefore, the search for other evaluation and treatment tools for these patients is an interesting challenge.

One powerful tool to enhance experimental control over such dynamic social interactions has been the use of avatars or virtual reality (Wu et al., 2014), and also the use of picture morphing interfaces (Fukuta et al., 2014). For example, a recent study shows that the use of virtual reality tools may present a number of advantages when dealing with patients suffering from schizophrenia, such as symptom assessment, identification of symptom markers, or the investigation of the differential prediction of symptoms (Freeman, 2008). It has also been shown that the use of virtual characters (avatars) can characterize, in a simple way, distortions in the social perception of patients (Kim et al., 2007). Other studies show that the distortions of reality and memory can be assessed using virtual environments (Sorkin et al., 2008; Weniger and Irle, 2008). This type of environments has also been applied as a therapy for conversational training programs in patients with schizophrenia (Ku et al., 2006, 2007).

Focusing on the emotional recognition through facial expressions, the use of avatars has a number of advantages over conventional methods based on static images, as they allow including the dynamic aspect of the facial expressiveness, as well as the control and parameterization of the expression timing and intensity, or the symmetry level and the duration of each expression. Therefore, the use of avatars could provide an efficient tool for the diagnosis and treatment of deficiencies in social and emotional behavior. However, while avatars are well suited to study human cognition (Joyal et al., 2014), there are still many open questions concerning both the theoretical aspects of employing virtual avatars in psychology and their practical application (Aymerich et al., 2014). As such, further investigation needs to be done in order to assess the suitability of employing virtual avatars in schizophrenia diagnosis and treatment.

### Experimental Goals and Hypotheses

The use of virtual agents constitutes a potential intermediate link between standardized tests and real-life functioning, and also offers the possibility of being used as an innovative medium for cognitive remediation (Oker et al., 2015). As such, the objective of the present study is to explore the possibilities of using a realistic-looking avatar previously introduced in Marcos et al. (2010) for the assessment of emotion recognition deficits in patients who suffer schizophrenia.

Although they are out of the scope of this pilot study, some of the benefits of an avatar over static images include: to allow the parameterization and fine tuning of the shown expressions; or to correlate the different expression intensity, timing and other characteristics of facial expressiveness with the patient recognition rates (RRs) and psychopathology.

In this context, we compared the response of a group of patients with schizophrenia and a control group using both a classical emotion recognition test based on static images, and a new test based on a dynamic avatar. Considering previous studies (Ku et al., 2006, 2007; Dyck et al., 2010) there is evidence that schizophrenia patients would also show deficits in the recognition of emotional expressions displayed by a virtual avatar. As such, our first hypothesis was:

*Hypothesis 1: Compared with controls, patients will show worse recognition scores, regardless the type of stimuli (avatar or images)*.

The benefits of dynamic facial expressions have been discussed lately. Some studies suggest that the dynamic properties of human facial movements may play a surprisingly small role in people’s ability to infer the emotional states of others from their facial expressions (Gold et al., 2013).

Others show that there is no difference in the use of dynamic or static images as a combination of hypersensitivity to static emotions and hyposensitivity to dynamic emotions in people with schizophrenia might underlie the absence of differences in response to these stimuli (Fukuta et al., 2014).

On the other hand, there is also evidence that the use of dynamic facial expressions, apart from being ecologically more valid and therefore more appropriate to emotion research, helps in the perception of emotions (Krumhuber et al., 2013). Based on the above assumptions, the second hypothesis we wanted to contrast was:

*Hypothesis 2: The avatar would turn out to have better recognition scores than static images*.

Finally, many studies have related the facial emotion recognition impairments of schizophrenia patients with their *psychopathology* (Dyck et al., 2010; Gold et al., 2013) and others. For that reason, our third hypothesis is:

*Hypothesis 3: If present, recognition deficits for the patients group are correlated with the psychopathology*.

## Materials and Methods

For the experiment, both patients and controls were asked to enter an office individually and to take a seat at a desk which held a computer screen. The therapist then explained them that some facial expressions corresponding to the six basic emotions: joy, sadness, fear, anger, surprise and disgust were going to be shown on the screen. It was also explained that expressions were going to be performed by an avatar that will start in a neutral pose or be shown in a static image, and that the goal was to try and guess the expression shown. The therapist insisted on the fact that there were no good or bad answers, and that the main purpose of the test was to collect the participants’ perception. The therapist then asked the participants if they had understood the instructions for the test, repeating them in the case the participant did not. The therapist tried to maintain a neutral tone of voice throughout the experiment, in order not to influence the responses.

**Figure 1:**
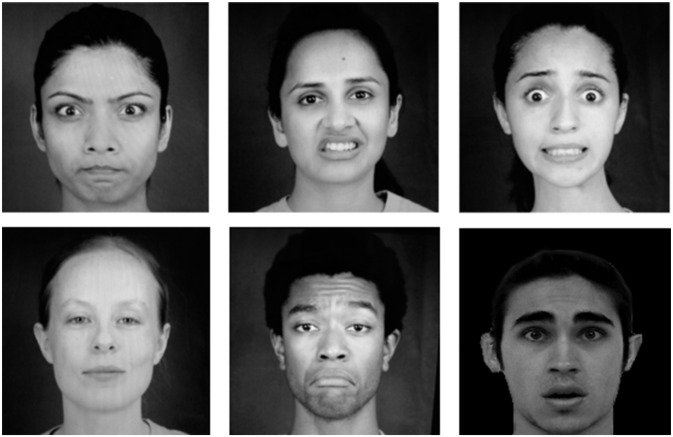
**The chosen static emotions**.

When the participant was ready, the therapist showed the different emotional expressions on the PC screen. Both the static images and the avatar were interspersed randomly and presented to the user, and the therapist waited up to 20 s for a response to be given. Images showed a static emotional expression (see Figure 1). On the other hand, the expression performed by the avatar started from a neutral pose and ended in the expression maximum. Avatar maintained the maximum of the expression until the therapist moved onto the next expression either because of a response given or if he considered a timeout. Correct answers were labeled as 1, whereas both wrong or lack of answer were labeled as 0.

### Subjects

Participants in the study consisted on two groups: patient and control. The patient group consisted of 20 subjects treated for schizophrenia at the San Luis hospital (Palencia, Spain). On an average, they are middle-aged patients and with a long evolution of the disease. After the research team checked that their cognitive level allowed them to understand instructions and the purpose of the test, they were asked to voluntarily participate in the study. The control group consisted of 20 subjects randomly recruited among healthy workers of the same center. Table 1 contains a survey of the demographics of both groups. Symptomatology was assessed by version A of the Positive and Negative Syndrome Scale (PANNS; Kay et al., 1987).

**Table 1:** **Demographics of both control and patient groups**.

	Age		Sex		PANNS positive		PANNS negative		PANNS psychopatology		PANNS total	
	Mean	S.D.	Male	Female	Mean	S.D.	Mean	S.D.	Mean	S.D.	Mean	S.D.
Control	42.7	10.8	7	13	–	–	–	–	–	–	–	–
Patients	55.0	8.3	11	9	21.8	7.5	27.4	9.3	47.1	11.3	97.0	27.8

### Materials and Equipment

#### Static Images

For the static images, six photos from the Paul Ekman model reflecting six universal emotions performed by different actors were selected. Images were selected so that the shown expression was visually similar to the one performed by the avatar, but also looking that the actors that performed them were from different races and genders in order to avoid biased results. Figure 1 shows the selected images for the different expressions.

#### Avatar Interface

The developed avatar follows a bio-inspired design looking for realistic results and consisting of two major steps: *modeling* and *animation*. The modeling step is carried out using a laser scanner that allows three-dimensional information of a real face to be obtained. Measured points are then meshed in order to obtain a 3D surface model. Face texture (color) is obtained from separate photographs that are mapped onto the 3D surface. This allows a degree of realism higher than that provided by other modeling methods to be achieved. Certain elements (eyes, teeth, oral cavity) have been modeled separately and then included in the whole model, since they could not be scanned.

**Figure 2:**
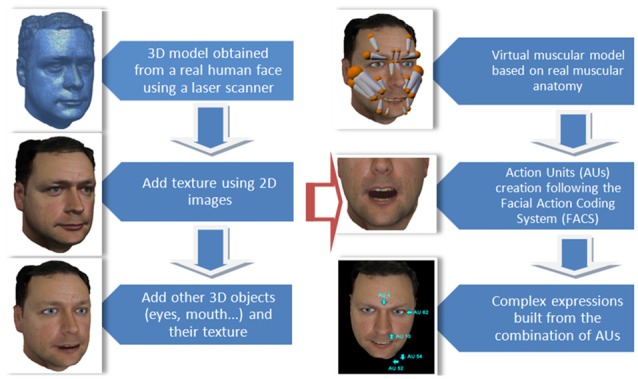
**Stages of the process of construction of the avatar.** Modeling (left) and animation (right).

The animation has been done through *pseudo-muscles* grouped into a *hierarchical skeleton*. The pseudo-muscles achieve a good approximation to the real facial muscles and have the advantage of requiring a lower computational effort, which makes them ideal for interactive real-time applications. The pseudo-muscle animation process has been carried out taking into account both the actual muscular physiognomy and anatomy. Once positioned, the pseudo-muscles are used to properly bend the three-dimensional mesh. The subsequent deformation is also based on the anatomy of the human face in the regions of muscle insertions into the skin. Figure 2 shows a scheme of the different stages of the process of modeling and animation. A more detailed description of the avatar construction and development is given in Marcos et al. (2010).

**Figure 3:**
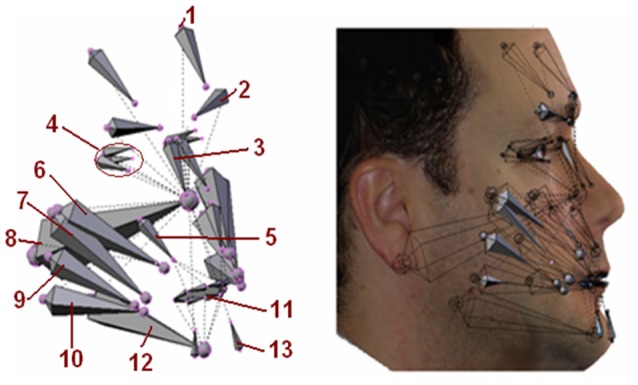
**Pseudo-muscles used in the avatar.** 1. Front; 2. Corrugator supercilii; 3. Procerus; 4. The eye muscles and orbicular of the eye; 5. Quadratuslabiisuperioris; 6. Zygomaticus major; 7. Zygomaticus minor; 8 Neck; 9. Buccinator; 10. Giggly; 11. Orbicular mouth; 12. Jaw; 13. Triangular from beard.

Figure 3 shows the set of pseudo-muscles used and their position on the virtual face.

**Figure 4:**
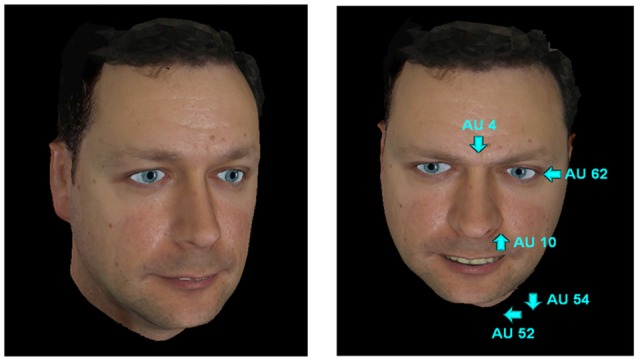
**Neutral expression and anger expression within the action units (AUs) of the facial action coding system (FACS) that generate it**.

The action of these pseudo-muscles produce deformations on the virtual 3D model in the same way as real facial muscles do on the face surface. The pseudo-muscle movements are based on the minimum action units (AUs) from the FACS. Complex expressions can be generated through the combination of AUs with different intensities, as shown in Figure 4.

**Figure 5:**
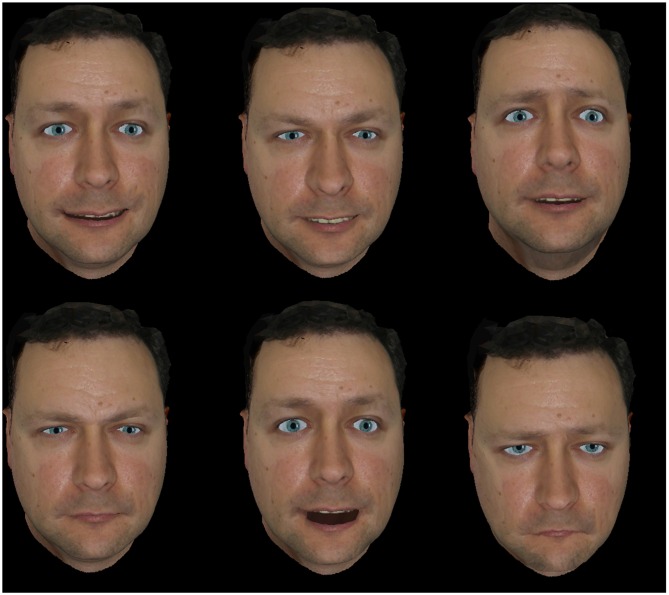
**The six emotional expressions generated by the avatar.** From top to bottom and from left to right: happiness, disgust, fear, anger, surprise and sadness.

For this experiment, we have generated six basic dynamic expressions: happiness, disgust, anger, fear, sadness and surprise (See Figure 5).

Also, the control interface of the avatar has been simplified in the current study, in such a way that the therapist only has to press a keyboard key to make the avatar perform a given expression. Although variables like intensity and timing can be modified, they have been kept constant in order to standardize the expressions shown to the participants of the experiment.

### Procedure

The study was carried out individually for each participant. Facial stimuli in the form of static images and real-time dynamically generated avatar expressions were presented to each participant, through a computer screen, in a random order. In total, participants were asked to identify the emotional expressions exhibited by six static images and six avatar expressions, each corresponding to the six universal emotional expressions: anger, fear, disgust, surprise, sadness and happiness.

Responses were recorded as successful if the emotional expression is correctly identified or as a failure if the emotional expression is wrongly identified or no response is given for a 20 s time limit.

### Ethics

This cross-sectional study was approved by the Clinical Research Ethics Committee of the Hospital it was performed in. Informed, voluntary, written consent was obtained from the patients and controls after a thorough explanation of the procedures, in accordance with the Declaration of Helsinki.

## Analysis and Results

All analyses were performed using the software SPSS, with the alpha level at 0.05 for statistical significance, unless otherwise stated.

### Descriptive Statistics

**Figure 6:**
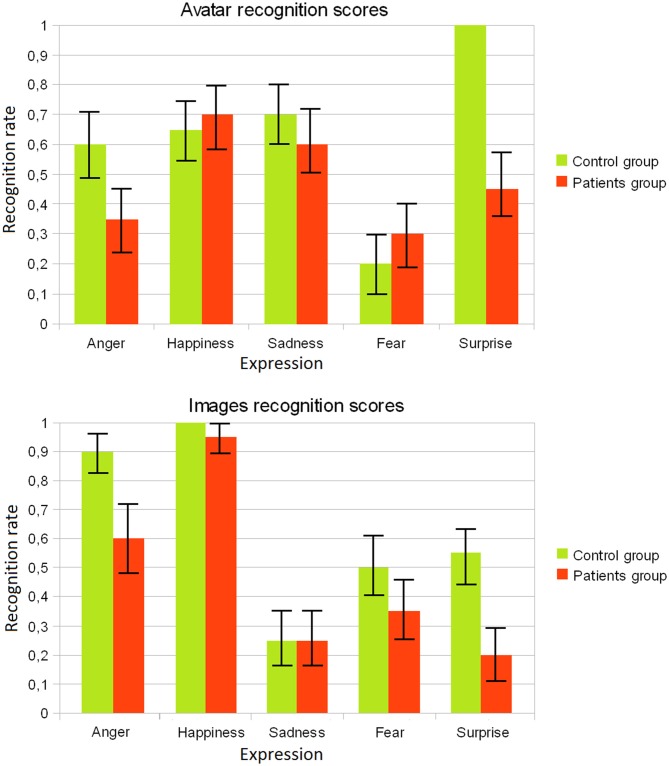
**Recognition rates obtained for the avatar and for the static images for each group (patients/controls)**.

In terms of descriptive statistics, a comparison of RRs for each of the emotional expressions for the two groups of participants has been done, by considering the responses obtained for the avatar against those obtained for static images. The RRs for the each group (patients/control) are shown in Figure 6. For the group of patients, it can be observed that the success rate for expressions of anger and happiness are higher for the static images, while expressions of sadness and surprise are more easily recognizable for the animated avatar. The expression of fear has similar RRs in both cases. For the control group, sadness, happiness and anger were better recognized in the case of images, while sadness and surprise expressions were better recognized in the case of the avatar. Finally the fear expression has a quite low RR.

It has to be noted that in the case of the expression of disgust, the expression generated by the avatar was not recognized by any participant, thus it was excluded from further analysis. We plan to rebuild this expression for future realizations.

Also, Table 2 shows a comparison of emotion recognition scores between both groups of participants. It can be seen that in the case of static images the RRs of the control group are better on average for all expression than those of the patient group. In the case of the avatar, however, control group only outperforms patient group in the case of surprise and anger expressions.

**Table 2:** **Mean and standard error for the different emotions**.

	Control		Patients	
	Avatar	Images	Avatar	Images
Anger	0.6/0.112*	0.90/0.069	0.35/0.109	0.60/0.112
Happiness	0.65/0.109	1/0	0.70/0.105	0.95/0.050
Sadness	0.70/0.105	0.25/0.099	0.60/0.112	0.25/0.099
Fear	0.2/0.092	0.50/0.115	0.30/0.105	0.35/0.109
Surprise	1/0	0.80/0.092	0.45/0.114	0.20/0.092

*Mean/SE.

### Recognition Rates Averaged for All Emotions

The answers of the participants were coded as a binomial variable labeled as *recognition* (recognition success/recognition failure), which in turn was considered as the dependent variable of our study. A set of bivariate analysis was first conducted in order to explore the relationship between the dependent variable and the other factors: *group* (control/patients), *face type* (avatar/images), *sex*, *age* and *emotion* (happiness, sadness, anger, fear, surprise). Table 3 surveys the results of the different tests for each considered factor. Simple binary logistic regressions were employed for the categorical variables, whereas a Student’s *t*-test was employed for the continuous *age* variable. Independent samples *t*-test showed no significant age differences between groups. As Table 3 shows, variables *face type* and *sex* did not result statistically significant (*p* = 0.614 and *p* = 0.212 respectively for the Wald criterion). On the other hand, statistically significant results were obtained for the variable *group* (*p* < 0.001) with an OR = 0.466 when considering the control group as a reference, indicating that the RR of the control group is overall two times higher than the one of the patient group.

**Table 3:** **Results of individual tests for the considered factors**.

Independent variable	*P value	**OR	***CI (95%)	
			Inf.	Sup.
Group	<0.001	0.466	0.311	0.698
Face type	0.614	1.107	0.745	1.645
Sex	0.212	1.288	0.865	1.917
Age	<0.001	4.6	0.405	6.781
Anger****	1	1	–	–
Happiness****	0.003	2.983	–	–
Sadness****	0.04	0.518	–	–
Fear****	0.001	0.322	–	–

*Wald criterion for dichotonomous variables, t-test for continuous variable age. **Odds Ratio (OR) for dichotonomous variables, mean difference for continuous variable age. ***Confidence interval (CI). ****Evaluated using Surprise emotion as reference.

Statistically significant results were also obtained for the *emotion* variable. In this case the *emotion* variable is polytomous, so in order to evaluate a regression model, one of its values is considered as a reference (e.g., surprise), while the rest are considered as “dummy” binomial variables, allowing to compute their OR. It can be seen that considering the surprise expression as a reference, there result statistically significant values for all expressions but anger, indicating that *emotion* has a significant contribution to the RR. In the case of *age*, statistically significant results were obtained for the *t*-test (*p* < 0.001). A further logistic regression analysis was performed for evaluating the effects of age over recognition, and using *age* as independent and non-categorical covariate. Results indicated that age indeed had a significant effect over the RR (*p* < 0,001 for the Wald criterion) and an OR = 0.950, which indicates that recognition success decreases with age.

Further explorations showed no statistically significant interactions between covariates. To increase the power of the statistical model, non-significant covariates were excluded for the analysis. Averaged across all emotions, a logistic regression analysis was then conducted using *age, emotion* and *group* as predictors, but the resulting model indicated a weak relationship between prediction and grouping (Nagelkerke’s *R*^2^ = 0.214), and overall prediction of 69.8% (74.9% for recognition success/63.0% for recognition failure).

### Recognition Rates for Each Emotion

Separated logistic regression analyses were then conducted for each emotional expression (see Table 4 for a survey of the results). In the case of anger, simple bivariate logistic regression analyses showed a strong relationship between the dependent variable *recognition* and *face type* (*p* = 0.013 for the Wald criterion), *group* (*p* = 0.013), and *age* (*p* = 0.003). No statistically significant interactions between covariates were found. Logistic regression conducted using those three factors produced a statistically significant model (chi square = 19.33, *p* < 0.001 with df = 3). Nagelkerke’s *R*^2^ of 0.291 indicated a weak relationship between prediction and grouping. Predictions success overall was 72.5% (83.7% for recognition success and 54.8% for recognition failure). The Wald criterion showed that only *face type* and *age* made a significant contribution to the model. The OR (Exp(B)) indicated that success in recognition decreased with *age*, and that participants were 4161 times more likely to recognize anger expression in the case of images. However, due to the said weakness of the model, we conclude that in the case of anger the model is unable to correctly predict the recognition success.

**Table 4:** **Survey of the results for the individual emotions**.

Expression	Main effects	**OR	Significance
Anger	Face type (*p* = 0.013)	4.161	Higher RR* for images
	Age (*p* = 0.003)	0.78	RR decreases with age
Happiness	Face type (*p* = 0.006)	18.98	Higher RR for images
Sadness	Face type (*p* = 0.04)	0.156	Better RR in the case of the avatar
	Age (*p* = 0.003)	0.948	RR decreases with age
Fear	NO SIG	NO SIG	NO SIG
Surprise	Face type (*p* < 0.001)	0.2	Higher RR in the case of the avatar
	Group (*p* < 0.001)	20	Higher RR in the case of the control group

*RR, Recognition Rate. **Odds Ratio (OR) for dichotonomous variables, mean difference for continuous variable age.

In the case of happiness bivariate logistic regression analyses showed a strong relationship between the dependent variable and *face type* (*p* = 0.006, OR = 18.778). No other covariates or interactions were found to be significant. For exploratory reasons, age and group were also included in the model. The regression model was statistically significant (chi square = 14.738, *p* < 0.002, df = 3). Overall prediction success was of 82.5%. Wald’s criterion showed no significant contribution for *age* and *group*, but in the case of *face type* it showed a significant contribution (*p* = 0.006, OR = 18.98) indicating a significantly higher RR in the case of the images.

In the case of sadness only *face type* (*p* < 0.01) and *age* (*p* = 0.037) were found significantly related to the dependent variable and so were introduced in the model. The test of the full model against the constant only model resulted statistically significant (chi square = 18.890, *p* < 0.001 with df = 2). Prediction success was 72.5% (69.4% success, 75% failure) with a Nagelkerke’s *R*^2^ of 0.281. Again, the RR decreased with age (OR = 0.948 for a continuous variable). For the *face type* the OR using the avatar group as a reference was 0.156 indicating that the recognition success was significantly higher in the case of the avatar.

In the case of fear, bivariate analyses indicated no statistically significant relationship between the dependent variable and any of the covariates.

In the case of surprise, simple bivariate logistic regression analyses showed a relationship between the dependent variable *recognition* and *face type* (*p* = 0.041 for the Wald criterion), *group* (*p* < 0.001), and *age* (*p* < 0.001). No additional statistically significant interactions between covariates were found. Logistic regression conducted using those three factors produced a statistically significant model (chi square = 37.903, *p* < 0.001 with df = 3). Nagelkerke’s *R*^2^ of 0.512 indicated a moderate relationship between prediction and grouping. Predictions success overall was 80% (81.6% for recognition success and 77.4% for recognition failure). The Wald criterion showed that only *face type* and *group* made a significant contribution to the model. The OR (Exp(B)) indicated that recognition success is likely to be 5× higher in the case of the avatar, and that the OR is 20× higher in the case of the control group.

### Correlations with Psychopathology

Considering only the patient group, success in the recognition of emotions proved to be correlated with the positive degree of symptomatology according to the PANNS. A simple binary logistic regression across all emotions and for both the avatar and images using recognition success as dependent variable showed that it was significantly related with the PANNS total (*p* = 0.039 for the Wald criterion). The Odds Ratio (Exp(B) = 0.989) indicated that the RR slowly decreases as PANNS increases. Other bivariate analysis with the rest of the covariates showed no significant relation with the dependent variable. Individual analysis for each expression did not show significant differences in terms of PANNS scores.

## Discussion

Consistent with our hypothesis, descriptive results show that schizophrenia patients present deficits in the recognition of virtual emotional expressions when compared with the control group. As Table 3 shows, the odds ratio of the average RRs for the control group is almost twice as high as those for the schizophrenia patients. Also, averaged across all emotions and for both control groups, the regression model showed that differences in RRs between the avatar and the images were not statistically significant. These findings match those previously presented by Kim et al. (2007) and Dyck et al. (2010), showing a reduction in social perception for schizophrenia patients even when employing virtual characters.

For the other considered covariates, sex did not produce statistically significant effects over the RR, while age produced significant variations, slightly decreasing the RRs as age increases. In the control group, this effect is somehow expected as the effects of age over expression recognition displayed by virtual characters has been related in the literature to the fact that elderly people is less used to technology and virtual environments (Marcos et al., 2010). It has also been related with the fact that visual acuity tends to decline with advancing age or even to the fact that older adults have an attentional bias towards old-age faces (Campbell et al., 2015). In the case of the group of patients, further studies that relate age with other factors due to the evolution of the schizophrenia shall be done in order to properly address this effect.

Regarding the relationship between recognition deficits and psychopathology, our findings also reveal an expected association between symptomatology and emotion recognition, as RRs decrease as PANNS scores increase. However, no statistically significant differences were found between the natural images and the virtual ones, suggesting that this relationship is independent of the type of facial stimuli employed. These results match those of Fukuta et al. (2014), and could be related to a combination of hypersensitivity and hyposensitivity to static and dynamic emotions respectively.

However, in the case of our second hypothesis, the recognition scores obtained by the avatar do not outperform those obtained from the images as expected. Analyzing each emotional expression individually, for the anger expression statistically significant results were found between the groups of patients and control as well as when comparing results for the avatar and static images. In this case, the anger expression is better recognized when using static images and the control group clearly outperforms the patient group. In the case of happiness, statistically significant differences were found between face type, being the expression better recognized in the case of the static images. In the case of sadness, face type was again found to be a main effect over the RR along with age. It can be seen, however, that in this case the avatar clearly outperforms the static images. No statistically significant main effects were found for the fear expression. Finally, in the case of sadness, both the face type and the group of participants turned out to be statistically significant for the differences found in the RR.

As Kohler et al. (2003) states, in the case of images patients have more difficulty to recognize negative emotions such as anger, fear, or sadness. Although other studies have shown slight improvements in the recognition of negative emotions when using virtual faces (Dyck et al., 2010), in our case this is only true of sadness but in a higher degree.

It is also interesting to point out that patients outperform the control group in the recognition of the happiness expression when facing the avatar, while the control group greatly outperforms the patients group in the recognition of the virtual expression of surprise. These results need to be further explored in future realizations as, although not statistically significant, from a quantitative perspective differ those obtained by Dyck et al. (2010), where recognizing differences between control and patient group were not so pronounced neither for the static nor the virtual images.

All these differences between our results and those from other studies lead us to consider that the improvements in emotion recognition that can be achieved when using dynamic virtual characters for emotion expression generation greatly depend on various aspects. On the one hand, expectation plays a fundamental role in the case of emotion recognition. For example, some studies show that there is a maladjustment of expectation when patients face social and nonsocial agents (Billeke et al., 2015). Also, it has been shown that patients correctly infer non-social, basic intentions, but experience difficulties when inferring non-social superordinate intentions and both basic and superordinate social intentions (Chambon et al., 2011). Finally, the selected facial features modified in each expression (i.e., the AUs activated in our avatar and their intensity and their associated muscles) may also have an influence in the recognized expressions.

The present study has helped us to confirm that schizophrenia patients also show deficits in emotion recognition when emotions are expressed by avatars, and that those impairments are related with the psychopathology. Also, our results suggest that further studies need to be done in order to assess the different factors that influence the recognition deficits present in patients who suffer schizophrenia.

As such, virtual avatars could enhance other recent studies in social cognitive impairments in schizophrenia such as facial mimicry (Sestito et al., 2013), or virtual reality environments (Peyroux and Franck, 2014).

In relation to the disgust expression generated by the avatar, it was not correctly recognized by any of the participants. As such, although it was correctly recognized in the case of the static images, those results were excluded from the study. In fact, the avatar disgust expression was confused with anger, fear and even happiness, but participants never related that expression with disgust. This lack of recognition may be related to the fact that the main difference between our disgust and anger expression is due to AU10, which corresponds to nose wrinkle and upper lip rising. However, due to our avatar configuration, the nose wrinkle does not appear clearly, thus making it difficult to be appreciated. In the near future, we plan to rebuild some expressions of the avatar and refine others, towards obtaining a higher expressivity. Also, the possibility to quantitatively tune the intensity and timing of expressions, as well as including other interaction channels such as voice will be exploited for a more accurate diagnosis and training of patients’ psychopathology.

## Author Contributions

EG-P designed the experiments and helped to draft the manuscript. CM-L, EG and LAF carried out the studies, and participated in the data analysis and discussion from a psychiatric perspective. SM-P designed and programmed the interface, participated in the data analysis and discussion and helped to draft the manuscript. JG-G-B and EZ participated in the interface design, the data analysis and discussion and helped to draft the manuscript.

## Conflict of Interest Statement

The authors declare that the research was conducted in the absence of any commercial or financial relationships that could be construed as a potential conflict of interest.
