# Corrigendum: Virtual Avatar for Emotion Recognition in Patients with Schizophrenia: A Pilot Study

**DOI:** 10.3389/fnhum.2016.00554

**Published:** 2016-11-02

**Authors:** Samuel Marcos-Pablos, Emilio González-Pablos, Carlos Martín-Lorenzo, Luis A. Flores, Jaime Gómez-García-Bermejo, Eduardo Zalama

**Affiliations:** ^1^Cartif Foundation, Parque Tecnológico de BoecilloValladolid, Spain; ^2^Research Unit, Hermanas Hospitalarias Centro Sociosanitario PalenciaPalencia, Spain; ^3^Instituto de Tecnologías Avanzadas de la Producción, Departamento de Ingeniería de Sistemas y Automática, University of ValladolidValladolid, Spain

**Keywords:** schizophrenia, facial recognition of emotions, realistic virtual avatar

In the original article, we have neglected to thank our sponsors. The following Funding section should be added:

“This work was supported by the Ministry of Science and Innovation, fundamental research project ref. DPI2014-56500-R and Junta de Castilla y León ref. VA036U14.”

The authors apologize for this oversight. This error does not change the scientific conclusions of the article in any way.

## Author contributions

All authors listed, have made substantial, direct and intellectual contribution to the work, and approved it for publication.

### Conflict of interest statement

The authors declare that the research was conducted in the absence of any commercial or financial relationships that could be construed as a potential conflict of interest.

